# A randomized controlled trial for evaluating pain response in patients with spinal metastases following local versus whole vertebral radiotherapy: study protocol for phase II clinical trial

**DOI:** 10.1186/s12883-022-02746-7

**Published:** 2022-06-20

**Authors:** Li Yuan, Lidan Geng, Danfeng Wu, Tangzhi Dai, Gang Feng, Xiaobo Du

**Affiliations:** Department of Oncology, Mianyang Central Hospital, No. 12 Changjiaxiang, Mianyang, 621000 China

**Keywords:** Vertebral metastasis, Radiotherapy, Pain control

## Abstract

**Background:**

Patients with bone metastasis often experience severe pain that is difficult to control and seriously affects quality of life. Radiotherapy is an effective way to relieve pain in these patients. Currently, there is no standard recommended range of radiotherapy targets for vertebral metastasis. The effect of radiotherapy on pain relief varies among patients, and some patients with metastases have serious side effects.

**Methods:**

This study aims to verify whether reducing the radiotherapy range for vertebral metastases can achieve the same effect as whole vertebral radiotherapy while minimizing side effects. Sixty-six patients with pain caused by vertebral metastasis were randomly divided into two groups. The study group is receiving partial vertebrae body radiotherapy at the regions of abnormal signal, suspected invasion, and adjacent subclinical focus of vertebral metastasis, and the control group is receiving the same dose of radiotherapy on whole vertebrae body where metastasis occurred. After radiotherapy, along-term follow-up of patients will determine pain relief and side effects.

**Discussion:**

The expected results of this study are that local irradiation of vertebral metastases can achieve a palliative effect of pain control not less than total vertebral irradiation with fewer side effects.

**Trial registration:**

This study was registered in the Chinese Clinical Trial Registry (No: ChiCTR1900023401).

## Background

Patients with bone metastasis often present with severe pain that is difficult to control. The ASTRO guideline and its update recommend palliative analgesic radiotherapy for bone metastases [1, 2]. However, the effects of pain control therapies vary from one patient to another. In the past, the target setting of two-dimensional radiotherapy and 3D-CRT included the whole diseased vertebral body to treat spinal metastases, despite some patients suffering from pathological fracture, spinal cord compression, and other side effects of an excessive dose or range of vertebral irradiation.

**Fig. 1:**
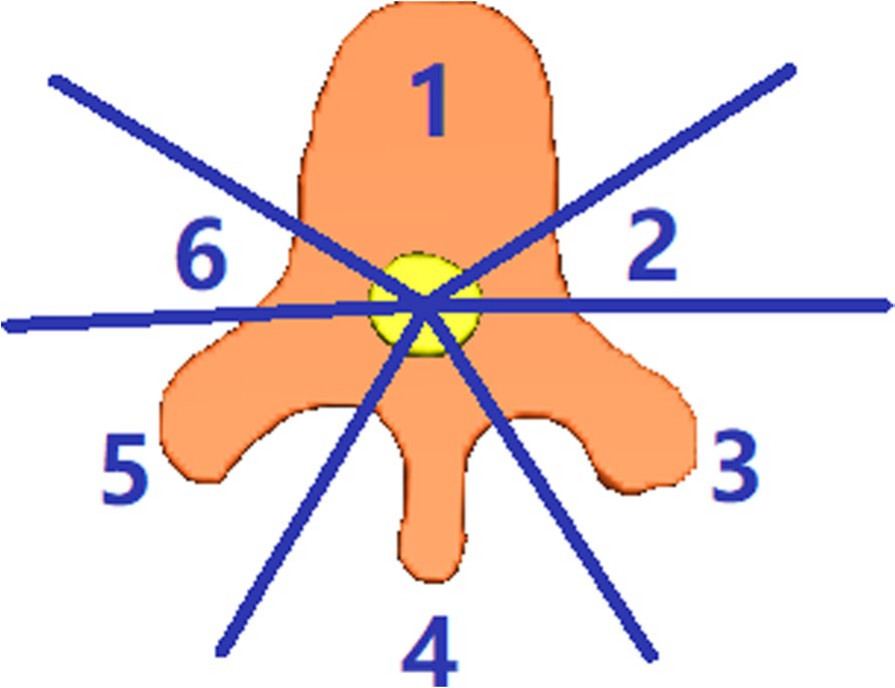
The International Spine Radiosurgery Consortium proposed to divide each vertebral body into 6 sectors: Sector 1 represents the vertebral body, sector 2 represents the left pedicle, sector3 represents the left transverse process and lamina, sector 4represents the spinous process, sector 5 represents the right transverse process and lamina, and sector 6 represents the right pedicle

The ISRC proposed dividing vertebral body into six parts in the target setting of spinal stereotactic radiosurgery, as shown in Fig. 1. As reported by a prospective study [3], there is a need to expand the CTV in SBRT for include adjacent physiologically occurring bone marrow spaces, which may have subclinical diseases and may become local recurrent lesions. The ISRC consensus guidelines on the target volume of spinal SRS suggest that CTV should include bone marrow signals suspected of microscopic invasion and adjacent normal bone. If the spinous processes, lamina / bilateral pedicle and vertebral body are affected or there are extensive metastatic diseases around the epidural space, the circumferential TVs around the spinal cord should be used [4].

At present, there is a single-center phase II prospective cohort study comparing the effect on pain control of conventional radiotherapy and SBRT in patients with spinal metastasis. In both treatment groups, patients had a comparable decrease in pain and analgesic use in the 3 months following treatment [2, 5]. However, there have no randomized controlled trials comparing the effect of local irradiation and whole vertebral irradiation on pain control in IMRT [6]. Therefore, we designed a randomized controlled trial to evaluate if irradiation of local metastases in the vertebral body as well as whole vertebral body irradiation can effectively control pain. We also investigated if side effects such as twinkling bone pain, spinal cord radiation injury, vertebral compression fracture, and nerve root compression were reduced.

## Methods

**Fig. 2:**
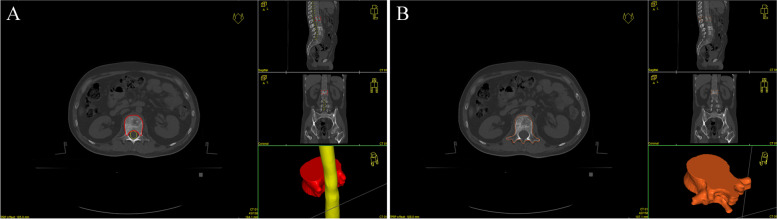
The experimental and control group clinical target volume setting (red line): **A** The clinical target volume setting of the experimental group was as follows: vertebral and adjacent tissue destruction according to the International Spine Radiosurgery Consortium consensus guidelines that recommend that the vertebral body is divided into six parts. **B** The clinical target volume setting of the control group: the entire vertebra that has been destroyed

This study was registered in the ChiCTR (No: ChiCTR1900023401). This is a multicenter, prospective, phase II randomized controlled clinical trial. Patients with solid tumors diagnosed pathologically at our hospital were collected. All patients had vertebral metastases and pain symptoms. Patients will be randomized into 2 groups: study group^’^s clinical target volume setting was according to the ISRC consensus guidelines that recommend the vertebral body be divided into six parts, which include the area of vertebral destruction and adjacent parts, and control group’s clinical target volume include the whole vertebral body, The two groups of clinical target volume settings are shown in Fig. 2. Two groups will receive treatment with the same dose of 95% PTV DT 30 Gy/10 f IGRT (Image Guided Radiation Therapy).

Before starting treatment, the qualified recruitment participants will explain the common ethical issues, objectives, design, description, implementation, requirements and schedule of this study to every potential study participant.

### Objectives

The primary endpoint is pain relief rate. The secondary endpoints are the incidence and types of side effects.

### Inclusion criteria

1. Aged 18-80 years
2. Patients with vertebral metastasis of solid tumors confirmed by pathology and imaging, and a number of discontinuous vertebral metastases ≤ 3, who had no tumor tissue compressing the spinal cord
3. ECOG0-1
4. No serious chronic diseases, such as severe hypertension, diabetes or heart disease
5. There was no contraindication to radiotherapy and no previous radiotherapy targeting a vertebral metastasis
6. Estimated survival time ≥ 3 months
7. The patient or their family members sign the informed consent form and were willing to cooperate with the follow-up

### Exclusion criteria

1. Bone destruction in benign diseases and diffuse bone destruction, such as multiple myeloma, lymphoma, or leukemia with bone marrow infiltration
2. Participated in other clinical trials of drug or physical therapy for target lesions in the past four weeks
3. Uncontrolled and/or severe disease, including:
  - Myocardial infarction or myocardial ischemia and arrhythmia (≥ 2 levels of congestive heart failure according to the NYHA and QTc ≥ 480 ms)
  - ≥ CTCA Elevel 2 infection or long-term unhealed wound
  - Active bleeding due to dysfunctional coagulation
  - Systolic blood pressure greater than or equal to 180 mmHg and diastolic blood pressure greater than or equal to 110 mmHg
  - According to the judgment of the researchers, the tumor invaded the large blood vessels and likely caused fatal bleeding during the treatment
4. Those who had a history of drug abuse and were unable to quit, or had mental disorders
5. Had received surgery or radiotherapy for the target lesion in the past six months
6. The researcher judged that there were serious accompanying diseases that would endanger the safety of patients or affect the completion of the study.

### Drop-out criteria

1. The withdrawal of the informed consent of patients.
2. A decision by the researchers to discontinue the study to ensure patients’ safety
3. A decision by the researchers or patients to stop the research in case of toxic side effects
4. Researchers’ judgment

### Radiotherapy

Before radiotherapy, all patients received computed tomography (CT) scans with a thickness of 3 to 5 mm. These CT scans were used to contour the gross tumor volume (GTV), clinical target volume (CTV) and organs at risk (OARs). The GTV was profiled according to CT scans, MRI and ECT. The clinical target volumes include the area of vertebral destruction and adjacent parts (study group) or the whole vertebral body (control group). The planning target volume (PTV) include organ movement and a daily setting error of 0.3 cm based on the CTV. Radiotherapy will be performed as IGRT, which will be administered using a line accelerator (energy = 6MV) in 10fractions (3 Gy/ fractionation, once a day from Monday to Friday).

### Data collection and management

This study will collect the patient’s name, age, gender, diagnosis of primary tumor, location and number of vertebral metastases, pain score and imaging findings. The above information will be recorded in a unique case report for each patient. These data will be input and saved in the electronic data management system established by Mianyang Central Hospital. The final data will be reviewed, locked and used for final data analysis by key researchers and statistical analysts.

### Measurement of the primary and secondary endpoints

Pain relief will be assessed by patients using the numerical rating scale before radiotherapy, two weeks, three months, six months and every six months after radiotherapy. The pain relief rate is the primary endpoint of this study. The secondary endpoint was a follow-up on the occurrence of side effects and the positive results of imaging, such as radiation spinal cord injury and pathological fracture. The vertebral bodies will be scanned with CT to identify radiogenic injury of the spinal cord and vertebral compression fracture.

Adverse events are being recorded in accordance with the requirements of the MedDRA. The classification of side effects was based on the CTCAE of National Cancer Institute.

### Statistical analysis and randomization

We estimated the sample size based on paper of Fleiss, JL et al. [7]. We performed non-inferiority and equivalence trials with α = 0.025, 1-β = 0.8 and non-inferiority *Δ* = -0.15 using Pearson*X*^2^test, power = 0.809, *H*0: πt—πc ≤ -0.15, *H*1: πt—πc > -0.15.We estimate a loss to follow-up rate of 10% and the resultant required number of patients was 66. The rate of pain relief in patients with vertebral metastases receiving radiotherapy will be measured according to the results of previous trials including that by Howell et al. [8]. Randomization will be administered centrally by the SPSS24.0 software at Mianyang central hospital. Patients who meet the inclusion criteria will be randomized on a1:1 basis to one of the 2 groups. The randomization will conduct by Mianyang Central Hospital through spss24.0 software. Patients who meet the enrollment conditions will enter the control group and the study group in a ratio of 1:1.

The number, proportion and 95% confidence interval of patients with pain relief will be reported. The related toxicity will analyze by Fisher exact test.

### Ethics

The study was approved by the Ethics Committee of Mianyang Central Hospital (NO 2019YJ30). The ethics committee will supervise and administer the study.

### Status

This trial was registered in the ChiCTR (NO: ChiCTR1900023401). The recruitment starts in January 2021 and is planned to be completed in two years.

## Discussion

Approximately one-third of patients with advanced malignant tumors will experience bone metastasis, and the spine is the most common bone metastasis, especially the lumbar spine and thoracic [9, 10]. Patients with bone metastasis often present with severe pain that is difficult to control. Some patients with vertebral metastasis even have pathological fractures and spinal cord compression. Radiotherapy is an effective treatment for alleviating the symptoms of bone metastasis [11–13].

The 2017 ASTRO update on palliative radiotherapy for bone metastases continues to recommend that patients with bone metastases can be selectively treated with 8 Gy/1 f, 20 Gy/5 f, 2 Gy/6 f, or 30 Gy/10 f with four different fractionation methods [14–16]. The 30 Gy/10 f and 8 Gy/1 f doses are widely used in conventional radiotherapy. Existing research has shown that the effects on pain relief of single radiotherapy and fractional radiotherapy are similar [11, 15–18]. However, Rades et al. showed that the recurrence of pain caused by vertebral metastases treated with large fractionation therapy could be as high as 26%, compared with conventional long-term treatment with 30 Gy/10 f, which showed better local control [19]. This is the basis for the selection of 30 Gy/10 F fractionated irradiation in our trial. We suspect that the reason for the difference in pain control and duration of pain relief may be related to the dose limitation of spinal nerves in patients with vertebral metastases. Therefore, current research on radiotherapy of vertebral metastases focuses on increasing the radiation dose without increasing the side effects to achieve better pain control [20].

According tothe2012 ISRC consensus guidelines on the target volume of spinal SRS, unless the spinous processes, lamina / bilateral pedicle and vertebral body are affected or there are extensive metastatic diseases around the epidural space, the circumferential TVs around the spinal cord should not be used. The guidelines emphasized that published guidelines on indications and practice patterns for spinal SRS are limited and there is no consensus on the definition of target volume [2]. We propose a safe and effective reduction of the target area in IGRT radiotherapy, which can effectively avoid the excessive irradiation of nerve, spinal cord and other normal tissues, and correspondingly improve the target dose setting.

If this trial can prove that local irradiation is equivalent to whole vertebral body irradiation in terms of pain relief, then the next step will be to increase the irradiation dose on the reduced target area in order to achieve higher pain control rate of vertebral metastasis and improve the quality of life of patients with vertebral metastasis.

## Data Availability

All data generated or analyzed during this study are included in this published article.

## Abbreviations

ASTRO: American Society for Therapeutic Radiology and Oncology
ISRC: International Spine Radiosurgery Consortium
3D-CRT: 3-dimensional conformal radiation therapy
CTV: Clinical target volume
SBRT: Stereotactic body radiation therapy
IMRT: Intensity-modulated radiation therapy
ChiCTR: Chinese Clinical Trial Registry
NYHA: New York Heart Association classification
IGRT: Image-guided radiation therapy
ECOG: Eastern Cooperative Oncology Group
MedDRA: Medical Dictionary for Regulatory Activities
CTCAE: Common Terminology Criteria Adverse Events
